# External Gas-Assisted Mold Temperature Control Improves Weld Line Quality in the Injection Molding Process

**DOI:** 10.3390/ma13122855

**Published:** 2020-06-25

**Authors:** Tran Minh The Uyen, Nguyen Truong Giang, Thanh Trung Do, Tran Anh Son, Pham Son Minh

**Affiliations:** 1 Faculty of Mechanical Engineering, HCMC University of Technology and Education, Ho Chi Minh City 71307, Vietnam; uyentmt@hcmute.edu.vn (T.M.T.U.); trungdt@hcmute.edu.vn (T.T.D.); 2 Department of Manufacturing Engineering, Faculty of Mechanical Engineering, University of Technology (HCMUT), 268 Ly Thuong Kiet Street, District 10, Ho Chi Minh City 72506, Vietnam; ntgiang.sdh19@hcmut.edu.vn (N.T.G.); tason@hcmut.edu.vn (T.A.S.); 3 Vietnam National University, Ho Chi Minh City 71300, Linh Trung Ward, Thu Duc District, Vietnam

**Keywords:** injection molding, thin wall injection molding, mold temperature control, external gas-assisted mold temperature control (Ex-GMTC), weld line appearance, weld line strength

## Abstract

Simulations and experiments were conducted with gas temperatures of 200–400 °C to investigate the impact of external gas-assisted mold temperature control (Ex-GMTC) on the quality of weld line of molding products. In the heating step, the heating rate was 19.6 °C/s from 30 to 128.5 °C in the first 5 s in a 400 °C gas environment. When applied to heating the weld line area of an injection mold, Ex-GMTC improved the appearance of the weld line when the cavity temperature was preheated to 150 °C. For the tensile strength test, a melt flow simulation comparing the packing pressure of different mesh thicknesses revealed that Ex-GMTC helped maintain a high pressure in the weld line area in different packing periods. This was verified by an experiment where Ex-GMTC was applied with 400 °C gas to change the mesh area temperature. The result indicated that an increase in the weld line area temperature from 60 to 180 °C improves the tensile strength of all mesh thicknesses, which was more pronounced with thinner parts, especially at 0.4 mm. The simulations revealed that high temperature is concentrated in the weld line area of the cavity surface, thus reducing the energy wasted during heating.

## 1. Introduction

Injection molding is a plastic fabricating process employed to achieve a wide range of product geometry, and is becoming increasingly popular in today’s industries due to its high efficiency and low-cost advantages. In this process, the raw material is first heated in an injection molding machine and transferred into a liquid melt. The hot melt is then pressed into the cavity for forming. When part of the volume cools down to the ejection temperature, the mold opens for product rejection. As a necessary step, the filling of hot melt into the cavity determines the part’s shape and the product quality depending on the temperature of cavity surface. With a high cavity temperature, the hot melt easily fills into the cavity, but the cooling step is longer and more serious warpage occurs. In the case of a low cavity temperature, the cooling time is faster, but filling the mold cavity with the hot melt becomes more difficult due to the development of a frozen layer [1].

Presently, customer demands for higher product quality and more complex product geometries have necessitated improvements in existing injection molding techniques. One of the effective methods used to address the issues encountered during the melt-filling step is mold temperature control [2,3]. For instance, molding of microproducts or parts with thin walls requires a high mold temperature. To meet the requirement of a high mold temperature during filling, substantial research has been conducted on many mold temperature control methods with the aim of increasing the cavity temperature while retaining a short cycle time. In the studies, the most popular solution was preheating the cavity surface prior to melt filling. This step raises the surface temperature higher than the glass transition temperature (Tg) of amorphous polymers or the melting point of semi-crystalline polymers; thus, with high cavity temperature, the speed at which the frozen layer develops is reduced, and the melt viscosity and flow resistance also decrease, allowing for an easier flow of the melt into the narrow cavity [4].

Several techniques were investigated to achieve the desired high temperature mold cavity. These include the application of hot water or hot oil, steam heating, resistance heating using cartridge heaters, and surface coating with a graphene heater. A common solution was applying a cooling channel with hot fluid flowing inside to raise the temperature of the mold plate to a target value. Flowing water was often used for target temperatures lower than 100 °C. Above 100 °C, either water flowed under high pressure or another fluid, i.e., hot oil or steam, was used [5]. Practically, both systems should operate under a high-pressure environment, which should be sustainable by the mold equipment.

For heating the mold to over 100 °C, electronic heaters were inserted into the volume of mold plate [6]. After that, the sheet heater was suggested for locally heating the mold surface. The result shows that the mold surface could be heated to 150 °C, and the frozen layer was reduced clearly [7,8]. However, the electronic heater system needs to be investigated for applying this method for the molding cycle. Therefore, the additional design as well as tool costs were required for this heating method.

For the mold temperature control described above, the volume of the mold plate is heated, implying that the heating source must be high enough to support the thermal energy for the mold plate. The cooling step begins as soon as the melt fills the whole cavity. Thus, in the cooling step, both the melt volume and the high-temperature mold plate need to be cooled, resulting in a longer cooling time. In general, the mold temperature method poses two challenges: waste of energy consumption and undesirably long cooling time. Accordingly, some studies [9,10,11] suggested a conformal cooling channel and demonstrated its ability to remove the thermal energy in the mold plate via simulation and experiment, especially for complex positions. Because manufacturing a conformal cooling channel is still dependent on 3D metal printing technology, the associated high cost, long manufacturing time, and strength of the mold plate remain challenges.

Surface temperature control methods were suggested to improve the heating efficiency and lessen the energy consumption during injection molding. These methods include heating by infrared, flame, laser, induction, or hot gas. With infrared heating, the initial mold temperature was set to 80 °C and the heating time to 10 s to allow the hot melt to complete filling into a microfeature [12,13]. Infrared heating increases the product strength, which increases the Young’s modulus and reduces the frozen layer [14]. With flame heating [15], the heating speed at the cavity surface is very fast; however, the mold structure, part geometry, and heat control remain issues when applied in injection molding, which is the reason for its rare use. In microinjection molding, laser heating is the choice for temperature control at the cavity surface [16,17] due to the extremely fast heating rate; however, heating by laser is only appropriate for small-sized products and requires additional equipment and coating of cavity surface. Induction heating, which applies the principle of electromagnetic induction, has been known over the last 10 years for its ability to quickly raise the cavity temperature of molds [18,19]. For injection molds, some of the types applied include heating by proximity induction [20], ring [21], and external coil [22,23]. Induction heating is known for its fast heating rate, and the heating process can be predicted by simulation. In addition, with external induction heating, the mold structure does not need an in-depth redesign. Conversely, temperature distribution, overheating at the edge of mold, and proper design of the heating coil for complex part geometry are some of its drawbacks.

Gas-assisted mold temperature control (GMTC) has primarily been applied for directly heating the mold surface [24,25]. In this method, hot gas flows into the mold cavity. Heat convection between the hot gas and the cavity surface increases the energy received by the mold surface, thereby raising its temperature. The hot gas entrance and exit system have to be inserted into the mold structure. Primary research conducted for GMTC revealed that the temperature of cavity surface could be increased to over 150 °C and, with a proper design for the heating system, demonstrated its ability to heat the complex cavity. However, one of the biggest challenges with the use of this method is that the mold structure must be redesigned relative to the requirements of the heating system, which often creates a highly complex mold structure for controlling the hot air flow. Recently, research showed that cavity temperature distribution with GMTC is not very good; the difference between temperatures could reach over 60 °C for a heating area of 80 mm × 40 mm. Thus, the application of GMTC for plastic injection molds still needs further study.

One of the defects observed in injection molding parts is weld line strength, which forms when two or more separate melt fronts traveling from different directions meet and join as the mold cavity is filled. In other words, weld lines reduce the mechanical properties of the product. Many studies were conducted to explain the weakness at the weld line and investigate solutions for improving the weld line strength. These results showed that certain molding parameters, such as melt temperature, mold temperature, injection speed, and packing pressure, have the most impact on the weld line strength [26,27,28,29]. For improving weld line performance, higher melt temperature, injection speed, and injection pressure were recommended [30,31]. In this research using polypropylene (PP), when the melt temperature increased from 200 to 240 °C and injection speed increased from 30 to 50 g/s, the visibility of the weld lines was reduced. However, according to these results, to improve weld line quality, stronger equipment, such as the injection molding machine, mold structure, and the cooling system, needs to be constructed. Some more solutions were suggested to improve the weld line strength, of which a high mold temperature was found to produce a positive effect [32,33]. However, the cooling step in this method is impractical as cooling the parts can be difficult and the temperature distribution may be undesirable, thus strongly affecting the warpage and molding cycle time.

To solve these problems, in this study, we applied local heating to the weld line area with the external GMTC (Ex-GMTC) system. The effectiveness of Ex-GMTC in improving the weld line quality of molding products during the injection molding cycle was investigated at varying gas temperatures of 200 to 400 °C. Separate simulations and experiments were conducted to evaluate the weld line appearance and the tensile strength of injection molding products. To study the weld line appearance, the effect of Ex-GMTC on a 0.4-mm-thick part was investigated with the heating area at the weld line position. For tensile strength, Ex-GMTC was applied at the center of the insert plate with varying mesh area thicknesses of 0.4–0.8 mm. After the molding process finished, the weld line of the molding product was observed to investigate the influence of Ex-GMTC on the weld line appearance. This heating method is applied to heat the mold surface of a mesh structure area, improving the product’s tensile strength. Simulations are conducted to observe the heating speed and temperature distribution. Experimentally, the temperature distribution of mold surface is researched by the Fluke TiS20 infrared camera. The experimental and simulation results were then compared for discussion.

## 2. Experimental and Simulation Methods

### 2.1. Experimental Method

**Figure 1:**
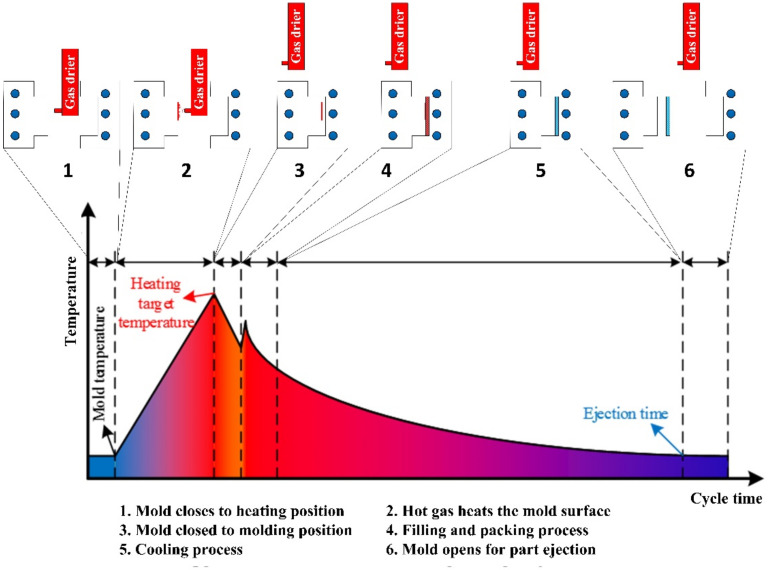
General steps of external gas-assisted mold temperature control in the injection molding process.

Considering the disadvantages of GMTC, in this study, Ex-GMTC was employed in injection molding for mold temperature control within a gas temperature range of 200–400 °C. The general process consisted of six steps, as presented in Figure 1. In Step 1, as soon as the molding cycle completed and the product was ejected, the hot gas source is transported to the heating area at which the cavity temperature is still low. In the next step, the hot gas sprayed directly into the heating area. Due to heat convection, the thermal energy was transferred to the cavity area, heating that area to the target temperature, after which the heating process stopped. At this time, the cavity remained at a high temperature. In Step 3, the gas drier was removed from the molding area and two half molds closed, before a new molding cycle began by melt filling into the cavity in Step 4. In this step, the hot cavity allowed the melt to easily flow into the thin-walled area. As soon as the entire cavity filled, the cooling process started with the heat transfer from the hot melt to the mold plate in Step 5. The process stopped when the part temperature reached the ejection temperature. In Step 6, the two half molds opened to eject the part, and the next molding cycle occurred.

**Figure 2:**
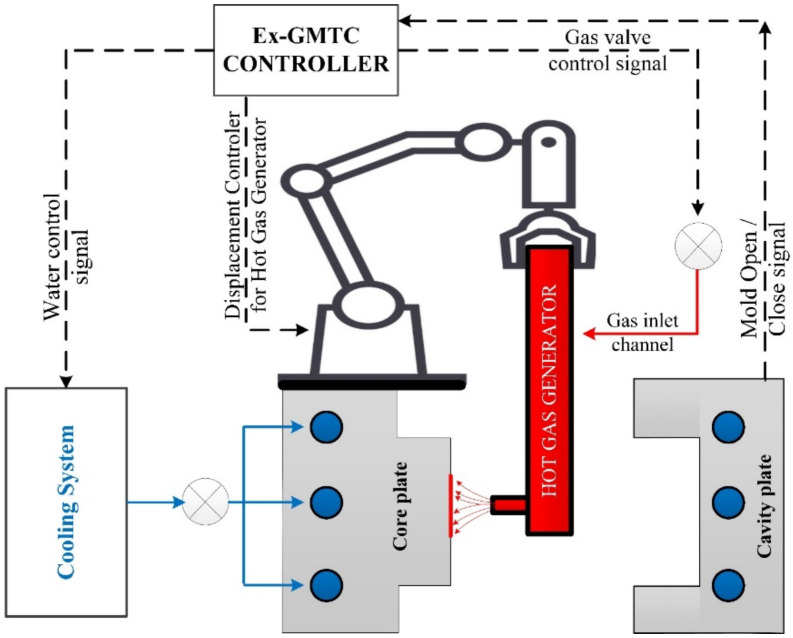
Schematic of the Ex-GMTC system.

Figure 2 shows the assembly of the heating system. This system includes a cooling system, a hot gas supporting with a 12-kW power, an Ex-GMTC controller. The controller will control the robot arm for moving the hot gas supporting. An air drier with outside dimensions of 240 × 100 × 60 mm was used to generate the hot air. Ambient air was allowed to flow into the air drier at 0.7 MPa, after which it flowed along the gas channel to absorb the thermal energy from the hot wall of air channel. The hot air then flowed out at the gas gate with a hole diameter of 10 mm. The high-power hot gas generator system supported the heat source, which provides a flow of hot air reaching 400 °C.

**Figure 3:**
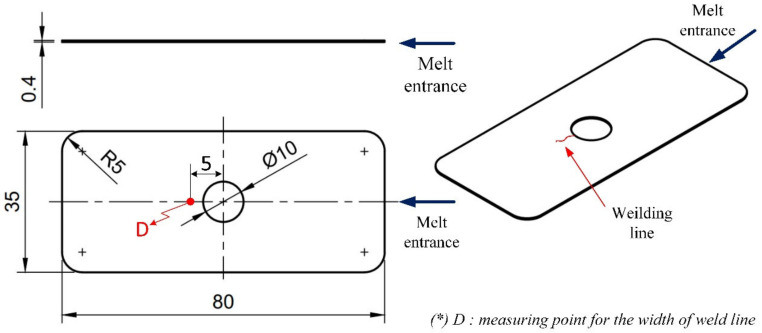
Dimensions of product used in the weld line appearance experiment.

**Figure 4:**
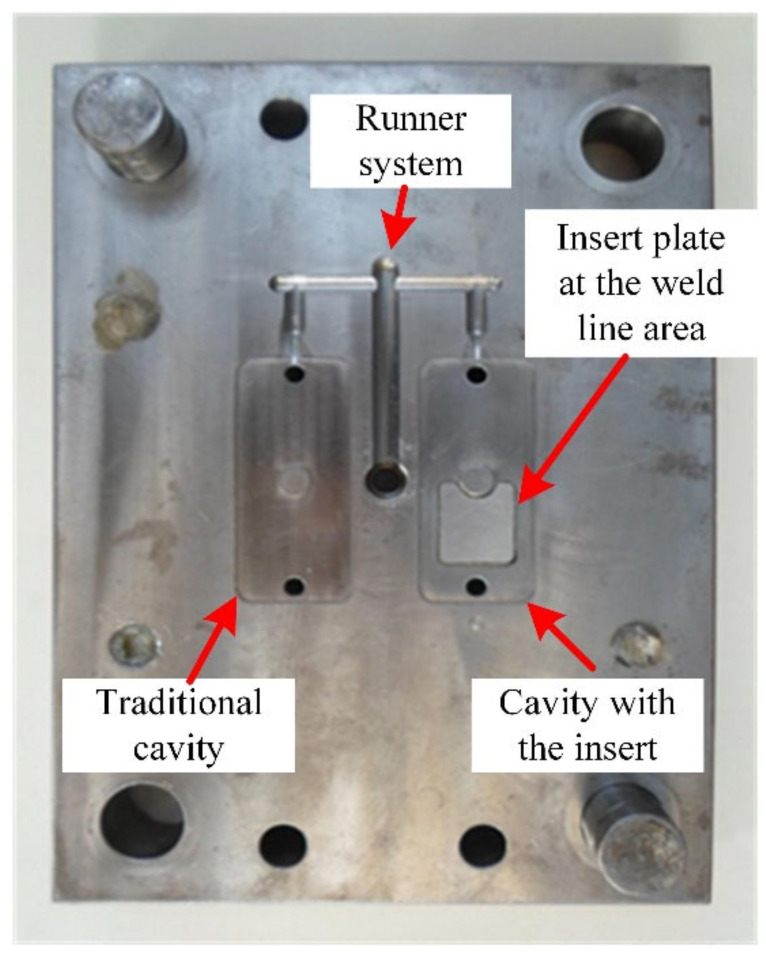
Mold design used in the experiment.

**Figure 5:**
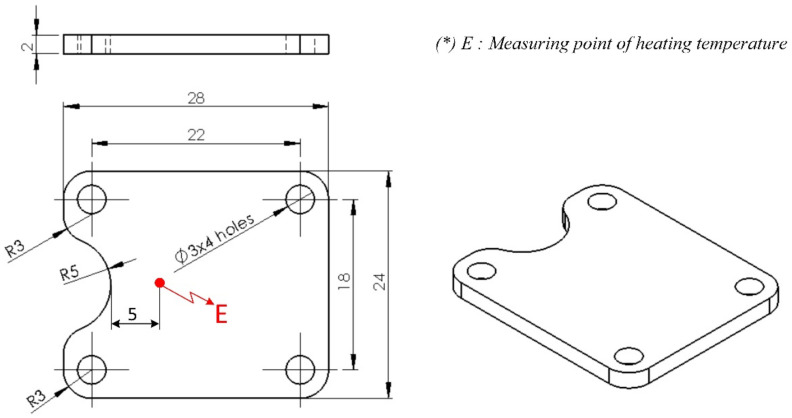
Insert plate used in the heating process.

A thin-walled product, presented in Figure 3, was examined to assess any improvement in the weld line appearance. This part had dimensions of 80 mm × 35 mm and a thickness of 0.4 mm; it also had a hole at the center. During injection molding, with the melt gate entrance presented in Figure 3, the hole was the main reason for the appearance of a weld line, with the two-flow molten plastics knitting together. The line creates issues in the product’s notch or gloss difference, discoloration, or glass fiber streaks in composite materials, which become more serious with thin-walled products. Figure 4 presents the injection mold design structure for the observation of the weld line. There were two cavities in the mold plate: one designed with a common structure and another with an insert for supporting the heating process at the weld line area, which is presented in Figure 5. This design is practical as it promotes heating efficiency and better control of the heated area.

**Figure 6:**
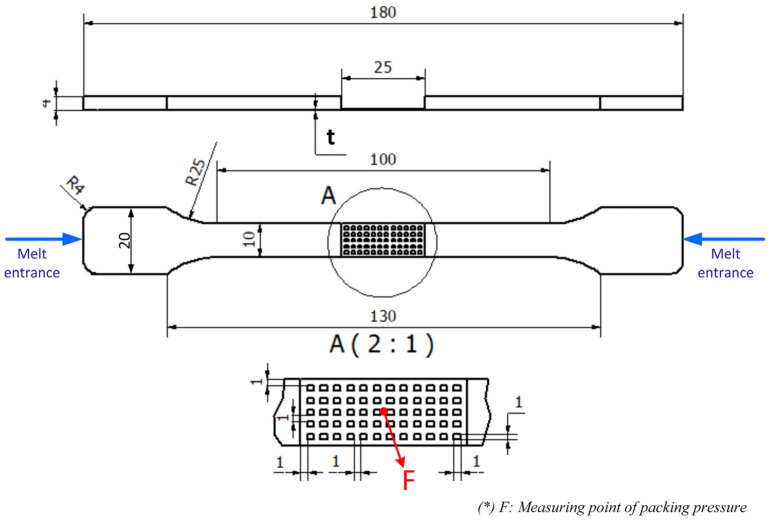
Dimensions of the specimen for a tensile strength test with a mesh structure at the center.

**Figure 7:**
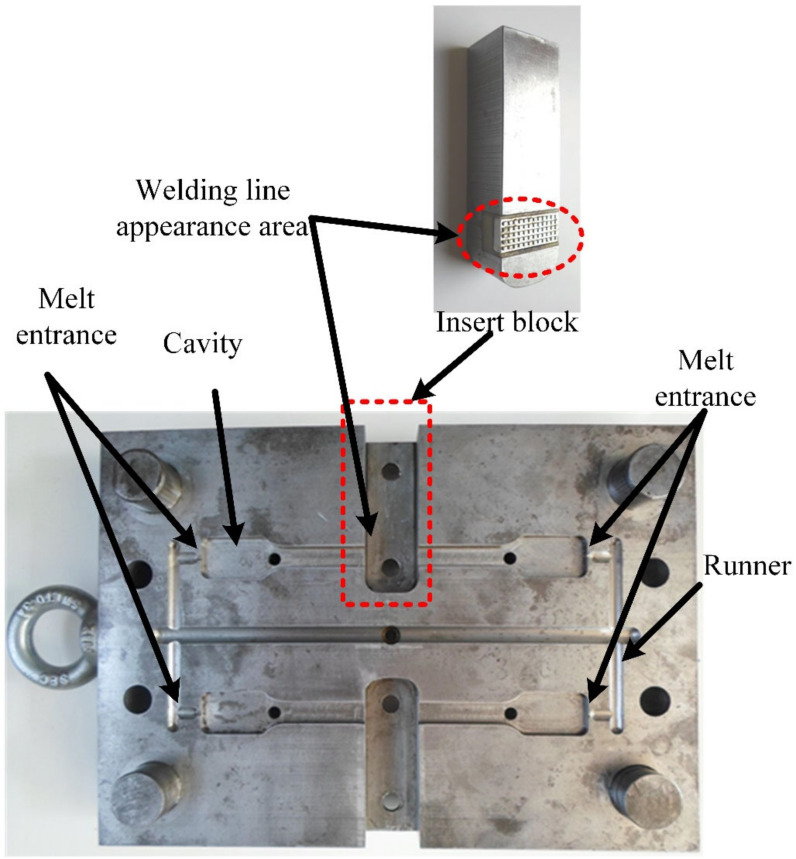
Cavity of the specimen used for the tensile strength test.

After investigation of the weld line appearance, the weld line strength was measured using a tensile strength test model. In some studies, a flat product was used for weld line strength measurement. In this study, the heating efficiency of Ex-GMTC on the complex cavity surface was verified using the model on a weld line area with a mesh structure. This design is detailed in Figure 6. This part was filled by two gates at the side, allowing the weld line to appear in the mesh area. The thickness (t) of the mesh area used was set to 0.4, 0.6, or 0.8 mm. To improve the tensile strength of the mesh structure, Ex-GMTC was applied to increase the temperature of the meshing area before the melt filled into this position. In the experiment, the tensile strength was measured with the mold presented in Figure 7, designed with two cavities. The melt filled both cavities; however, only one was heated by Ex-GMTC. Thus, the tensile strength could be compared both with and without Ex-GMTC. Each cavity was also filled by the two gates at the side, which allowed the weld line to appear in the center of the molding part.

**Figure 8:**
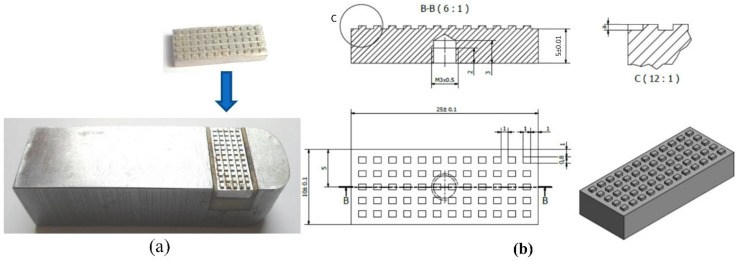
(**a**) block insert; (**b**) insert stamp.

As with the mold in Figure 7, the insert structure and stamp in Figure 8a,b were also applied in the mesh area. The insert stamp was 10 mm × 25 mm with a thickness of 5 mm. Here, the hot gas was sprayed directly to the insert stamp to increase the insert temperature and improve the quality of the weld line during the molding process.

**Figure 9:**
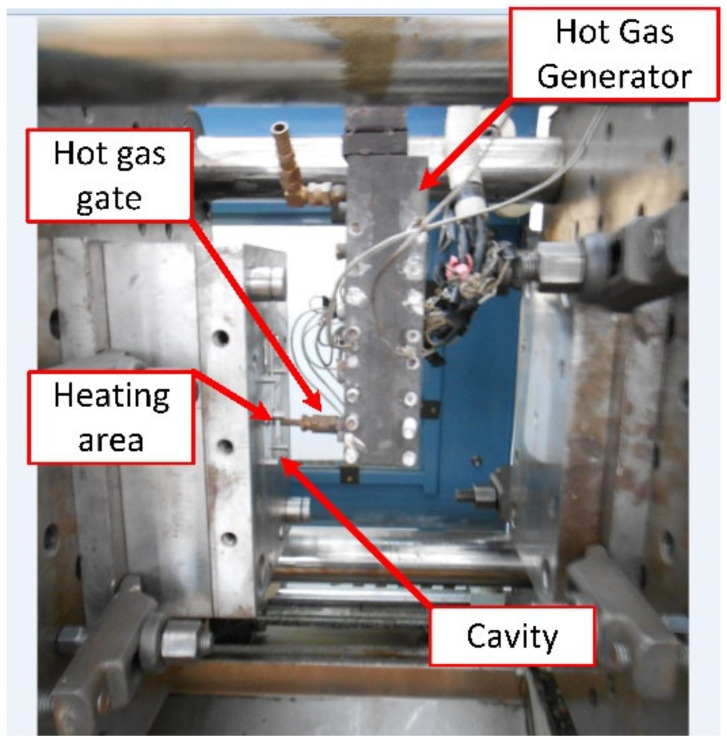
Heating step in the experiment.

In both experiments (weld line appearance and strength), the molds and the Ex-GMTC module were assembled using a SW-120B molding machine (Shine Well Machinery Co., Ltd., Tainan, Taiwan) and the other equipment shown in Figure 2. Figure 9 presents the heating step and the positions of the hot gas generator, hot gas gate, and heating area.

### 2.2. Simulation Method

**Figure 10:**
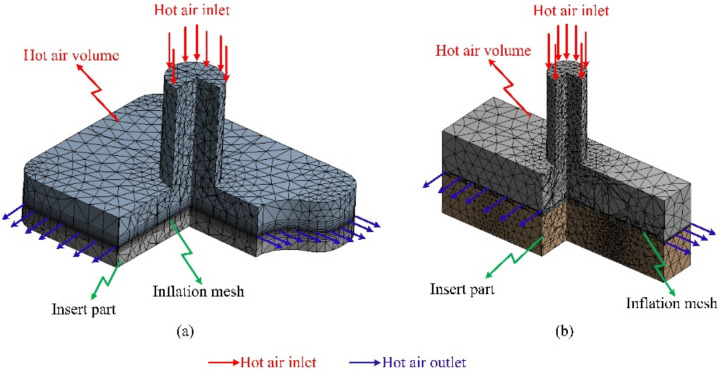
Simulation models for (**a**) weld line appearance and (**b**) weld line strength.

**Figure 11:**
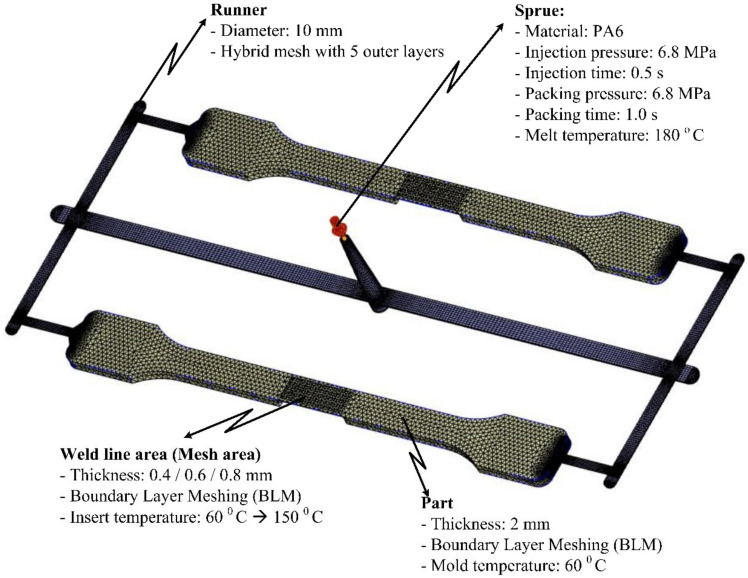
Meshing model and the boundary conditions of the packing simulation.

A simulation model was built to analyze the temperature distribution of the mold surface. Due to the structure of cavity insert in Figure 4 and Figure 8a, an insulation component was used to cover the heating area so that simulation models included only two parts: the insert part and the hot gas part. The boundary conditions of simulation and the meshing model are presented in Figure 10, and the simulation parameters are listed in Table 1. For increasing the simulation precision, the mesh of part insert was generated with a hex-dominant element. The inflation meshing method was applied with ten layers at the contact surfaces. Moreover, with the air volume, a tetrahedron element was used. The Ex-GMTC heating process was analyzed using the ANSYS software for the same experimental boundary conditions. In the simulation, the heat transfer mode around all external surfaces of the mold plate was set at free convection to the air, with ambient temperature of 30 °C and a heat transfer coefficient of 10 W/m^2^·K. Similarly, in the experiment, the area at the cavity center was designed with an insert plate to improve the heating efficiency. The temperatures of gas inlet used for the simulation ranged from 200 °C to 400 °C. As in many research works, the strength of the weld line was shown to depend on the packing pressure. In this study, the packing pressure at the mesh area was observed by simulation with the model presented in Figure 11 to understand the influence of mold temperature on the product strength. The Moldex3D software (CoreTech System Co., Ltd., Chupei City, Hsinchu County 302, Taiwan) was employed with the function of packing. The molding parameters were set up as in the experiment.

## 3. Results and Discussion

### 3.1. Effect of Ex-GMTC on Weld Line Appearance

Previous studies [23,24] reported an improvement in heating efficiency when internal GMTC is applied to raise the cavity temperature. However, this method is not suitable in some cases due to the different temperatures at the heating surface, as well as the need to improve the heating speed. In this research, for estimating the efficiency of Ex-GMTC in the mold heating process, the temperature distribution and the heating speed of cavity surface were used. Its impact on the quality of the weld line was investigated, with the mold designs in Figure 4 and Figure 7, in which the gas drier had one hot gas gate that mainly served to heat the cavity and improve the temperature distribution as well as the heating rate. The heating step was analyzed with the meshing model in Figure 10a with the hot air temperature varies from 200 °C to 400 °C and 30 s for heating time.

**Figure 12:**
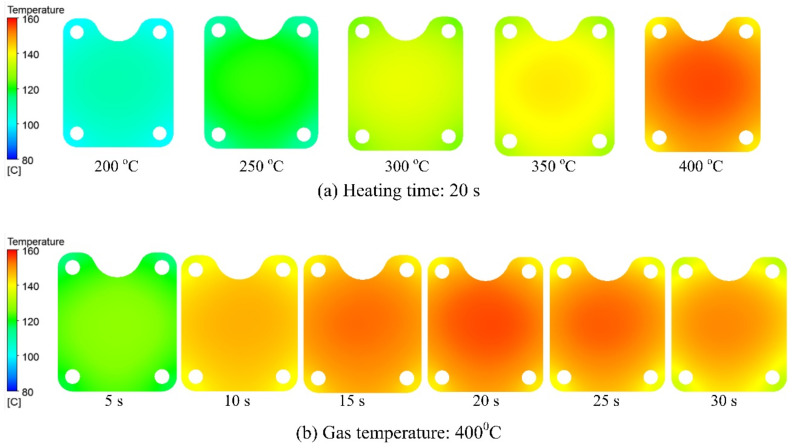
Simulation result of temperature distribution at the insert surface with changes in (**a**) heating source from 200 to 400 °C and (**b**) heating time from 5 to 30 s.

By simulation, Figure 12a presents the temperature distribution of the cavity surface at various gas temperatures with a heating time of 20 s. This result shows that the area near the hot gas gate has a little higher temperature, however, in that generated, the temperature distribution of cavity surface is almost uniformed. After 20 s, the cavity temperature reached a maximum at 158.7 °C, with a 400 °C gas; the minimum cavity temperature was 105.4 °C at a gas temperature of 200 °C. In Figure 12b, when a 400 °C gas temperature was employed, the temperature at the center of the cavity rapidly increased to 126.8 °C after the first 5 s of the heating process and eventually reached 158.7 °C at the end of 20 s, after which it dropped down gradually to 150.5 °C after 30 s. Note that the temperature difference were stronger at the starting of the heating step when the heating rate at the gas gate was still very strong. Such a phenomenon was also apparent at all gas temperatures. Likewise, this phenomenon also appears at the end of the heating period, mainly due to an imbalance in the thermal energy between the area near the gas gate and the distant area from the gas gate. When the higher gas temperature was used, the cavity surface exhibited a trend of releasing greater calorific power to the environment; therefore, in the area distant from the gas gate, the temperature was much lower than that of the area near the gas gate. Furthermore, the influence of the cavity insert was elucidated by the appearance of a higher-temperature area at the insert surface.

**Figure 13:**
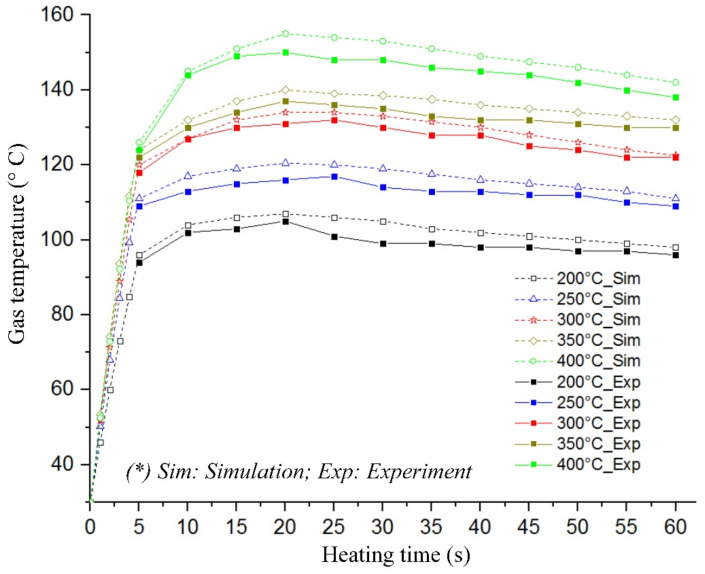
Heating process comparison between simulation and experiment.

The same boundary conditions were applied in the experiment to verify the simulation results. The temperatures at the insert plate were collected using a thermal camera (Fluke Corporation, Everett, Washington, DC, USA) and compared with the simulation results, as presented in Figure 13. The temperature was recorded at point E in Figure 5. Here, the temperature difference between the simulations and experiment was lower than 8.0 °C, which was mainly caused by a delay of infrared camera, especially as the thermal energy can transfer quickly from the hot area to the cooler one. However, generally, Figure 13 indicates a good agreement between the simulation and experimental values, and we experimentally identified the limitation in the heating process.

Figure 13 shows that the heating process with gas temperatures of 200, 250, 300, 350, and 400 °C and heating time of the first five seconds increased the cavity temperatures to these heating speeds: 13.6, 16.2, 18.4, 19.2, and 19.6 °C/s, respectively. Both the simulation and the experiment demonstrated a limitation on raising the temperature throughout the heating history. For instance, the heating efficiency was high only in the first five seconds of the heating step. In this period, the thermal energy of hot air contacts the insert, which was at low temperature; therefore, the heat transfer efficiency was highest. The heating result showed that the temperature at the measuring point (E) increased quickly in this step. However, as the temperature at the insert surface increases, the heat convection lowers. Thus, the insert temperature increased more gradually in the next 10 s; after 20 s, the cavity temperature remained stable. This phenomenon was reasonable due to the heat transfer from the hot gas to the cooler mold surface. In the heating process, an increase in the surface temperature would mean slower heat transfer. In this regard, for the five gas temperature values, high heating speed was achieved within the first 20 s, with the value of 19.6 °C/s and gas temperature of 400 °C, in this case, the insert surface reached 158.6 °C. This mold temperature was appropriately high for reducing the frozen layer of almost the plastic materials. On the contrary, this restriction of heating reduced a lot of trouble in the heating process, especially for the mold structure of micro part. This is also a benefit of Ex-GMTC against other heating methods for injection mold [14,15,16,17,18,19,20,21,22].

**Figure 14:**
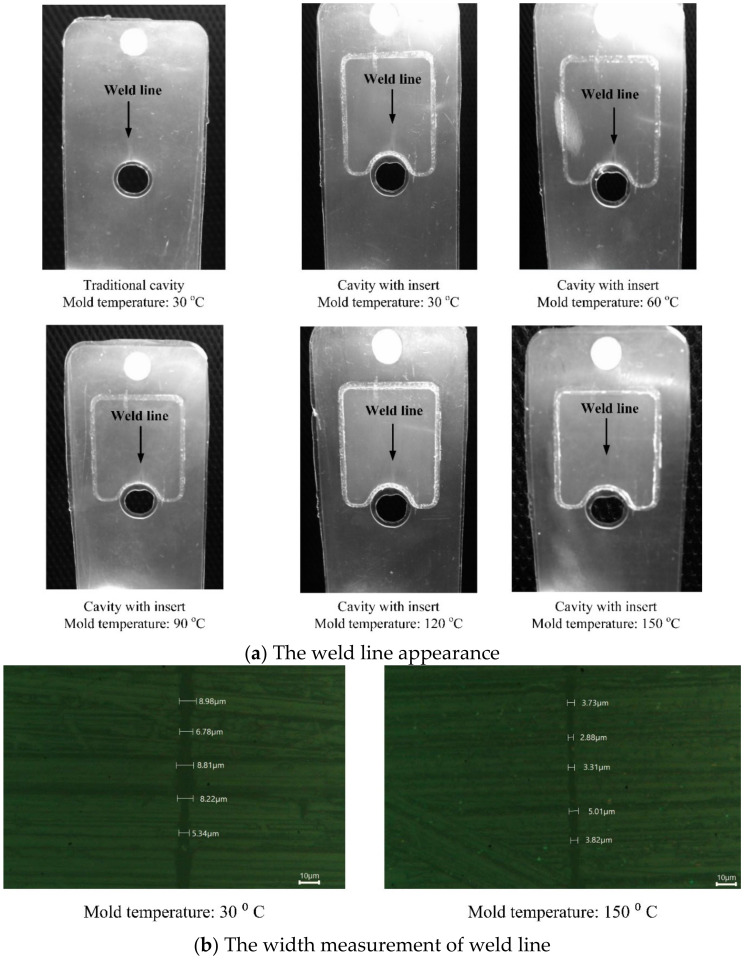
The comparison of weld line appearance (**a**) and weld line width (**b**) at different cavity temperatures.

To investigate the effect of hot gas heating in injection molding on the weld line appearance, an experiment was conducted with the molding parameters listed in Table 2. Polyamide 6 (Lanxess, Durethan B 30 S 000000, non-reinforced, ISO 1874-PA 6, GR, 14-030, Cologne, Germany) was used for this experiment. With the application of Ex-GMTC, the molding process was operated under different temperatures at the weld line position. Once the molding finished, the parts with weld line appearances were collected and evaluated, as presented in Figure 14a. Based on the results, the weld line appearance improved with the application of Ex-GMTC. The line became less obvious as the cavity temperature increased and almost disappeared at 150 °C. By using the Euromex Oxion Inverso Material Science Microscope (OX.2153-PLM, Arnhem, The Netherlands), the width of weld line was measured. The result showed that when the mold temperature increased from 30 to 150 °C, the width of the weld line decreased, as shown in Figure 14b. In detail, with a mold temperature of 30 °C, the weld line width varied from 5.34 to 8.96 µm. However, when the mold temperature increased to 150 °C, the width of weld line only varied from 2.88 to 5.01 µm. This can be explained by the reduction in resin viscosity at high temperature, which helped to achieve a better flow pattern and reduce cold welds at the positions where flow fronts met. Accordingly, this result also proved that the weld line appearance could be mitigated with a locally high cavity temperature; in detail, the mold cavity at the weld line position should be higher than 120 °C.

### 3.2. Effect of Ex-GMTC on Tensile Strength

**Figure 15:**
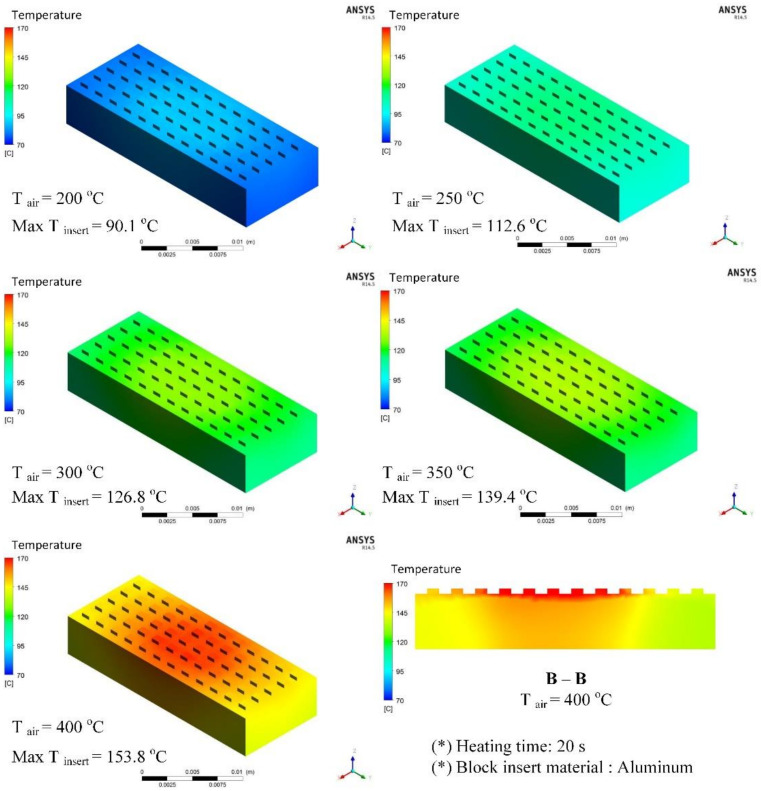
Temperature distribution of block insert with a heating time of 20 s.

Different types of plastic materials with different thicknesses were used for the simulation model presented in Figure 10b under a set analysis environment during heat transfer. Figure 15 presents the results of the temperature distribution for the block insert, which was good at a temperature difference lower than 5 °C. After 20 s of heating time, the minimum temperature achieved by the insert was 90.1 °C, whereas the maximum was 153.8 °C, positioned at the block center.

Figure 15 also shows that in the section B–B of the insert, high temperature concentrated on the surface of the insert plate at the position of the meshing structure for plastic products. This temperature distribution would facilitate the subsequent cooling process during the injection molding process. Practically, this is also one of the outstanding advantages of the hot air heating method. Based on the simulation results, depending on the temperature value of the hot gas flow, the cavity surface would reach equilibrium with different temperature levels.

**Figure 16:**
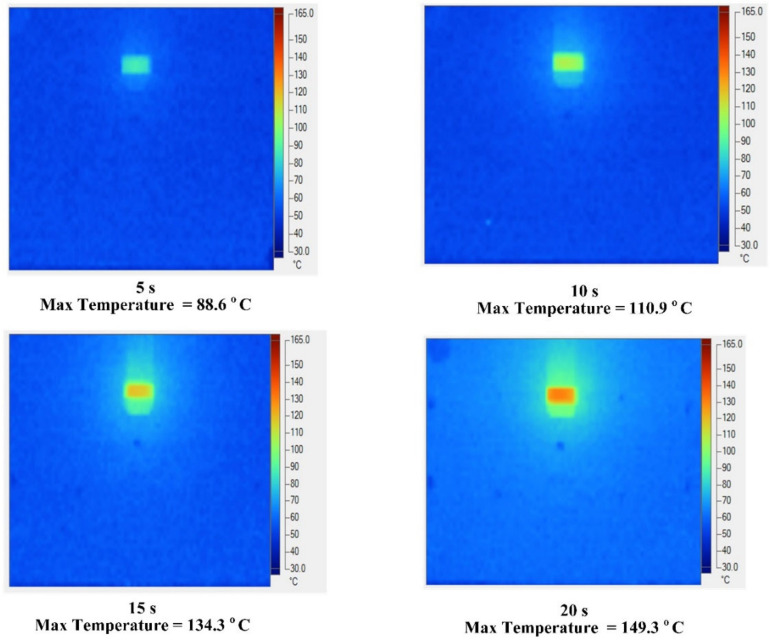
Temperature distribution of mold surface with a gas temperature of 400 °C.

To verify the simulation results above, an experiment with 400 °C gas was conducted to understand the temperature distribution. The data collected are presented in Figure 16. The maximum temperature was reached at the mesh area with values of 88.6, 110.9 134.3, and 149.3 °C under heating times of 5, 10, 15, and 20 s, respectively. Based on the distribution data, Ex-GMTC demonstrated very good local heating performance. High temperatures focused at the mesh location where the weld line appeared, whereas the temperature was low in other locations, which is one the advantages of the hot gas heating method (in particular) and the surface heating (in general). Thus, after heating and filling of melt in the whole cavity, the cooling step for the cavity becomes easier, with a very small high-temperature area compared with the entire mold plate volume. In terms of wasted energy, the temperature distribution at the cavity surface revealed that nearly all of the heat in the heating process concentrated on the area that required heating, which showed the effectiveness of the Ex-GMTC method in saving energy. This result also suggested that Ex-GMTC could be efficient for the complex cavity surface in Figure 6, which was not considered in previous studies [17,18,19,20,21,22,23,24,25].

**Figure 17:**
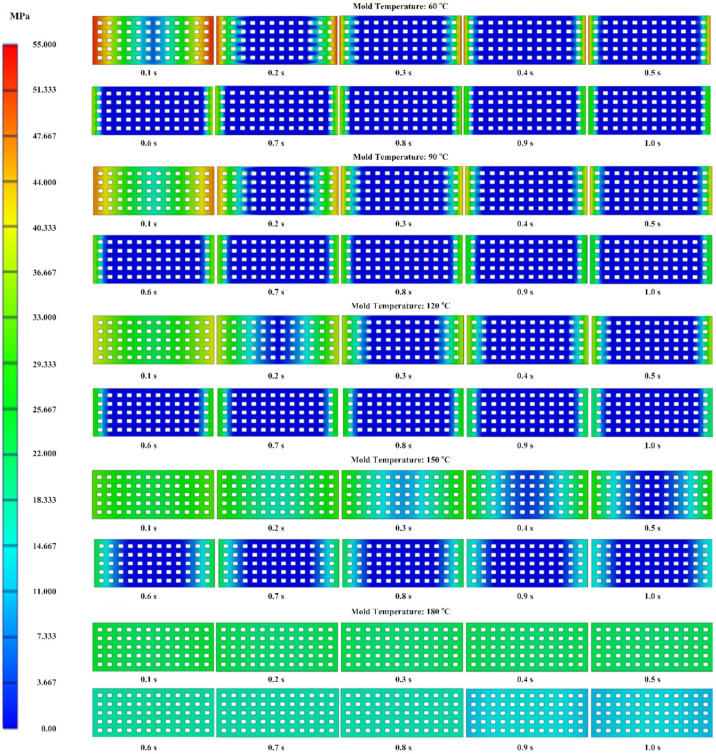
Pressure distribution at the weld line area with part thickness of 0.4 mm under different mold temperatures using PA6 material.

**Figure 18:**
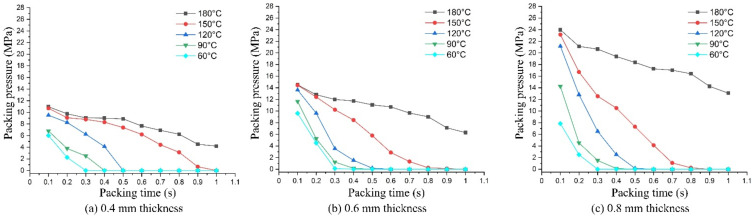
Packing pressure at the center point (F) of obtained products with PA6 material with part thickness of 0.4 mm (**a**), 0.6 mm (**b**) and 0.8 mm (**c**).

As mentioned earlier, in injection molding processes, the packing pressure affects the process of forming the product and the mechanical properties of the product after molding [31,32]. For this, a simulation and an experiment of the packing step were conducted with the filling of material PA6 based on the simulation model in Figure 11. The thickness at the mesh area was varied from 0.4 to 0.8 mm. The influence of Ex-GMTC on the packing pressure and the weld line strength was observed at mesh area temperatures of 60–180 °C. The change and distribution in packing pressure were investigated with Moldex3D simulation from 0.1 to 1.0 s. Distributions of the packing pressure and the pressure at the center point (C) were collected and then compared, as presented in Figure 17 and Figure 18.

Figure 17 presents the packing pressure at the mesh area with the 0.4 mm part thickness at various packing times of 0.1–1.0 s under different mold temperatures. At the beginning of the packing period for all temperatures, the pressure was high on both the left and right sides of the mesh area, while the pressure was lowest initially at the center. The low pressure expanded over time. A distinct change in the pressure distribution was observed for mold temperatures of 60–180 °C; at 60 °C, the low-pressure area almost fully appeared at 0.3 s, whereas, at high mold temperatures, the higher pressure was maintained until 1.0 s.

From the experiment, Figure 18 presents the definite change in the packing pressure at point F from 0.1 to 1.0 s for different mold temperatures and part thicknesses. In general, the higher the cavity temperature, the longer the packing pressure was maintained, which can be explained by the solidification of the plastic in contact with the cavity that creates the frozen layer. At high cavity temperature, the frozen layer formed more slowly, allowing the melt to remain in liquid form for a longer time and facilitating transfer of injection pressure from the injection molding machine to the mesh area. As a result, the high pressure at the weld line position was maintained longer than for a low cavity temperature. With respect to the variation in part thickness, a thinner product leads to faster reduction in the packing pressure mainly because a thinner flow allows a faster heat transfer between the melt and the cavity, whereas the melt solidifies faster with a thicker product. Nevertheless, when the heating step was applied to the cavity, the packing pressure remained high, especially for the product with a thickness of 0.4 mm. Based on these results, the gas heating method can be used for the cavity surface to positively affect the change in packing pressure. This is important for improving the strength of injection molding products.

Comparing the results between simulation and experiment, we found good agreement: the higher the mold temperature, the longer the packing time. However, we found a difference in the packing pressure between the simulation and experiment. This difference occurred because, in the experiment, the Ex-GMTC only heated the cavity side because the core side had the pressure sensor; to protect the sensor, the heating could not be applied to this side. The result was the frozen layer being reduced only on one side of the mold. Conversely, in the simulation, by setting up the mold temperature with the same value at the end of the heating step, both the cavity side and core side were run at high temperature. The result was the frozen layer decreasing on both sides. This is the main reason that the packing pressure in the simulations was always higher than in the experiments.

**Figure 19:**
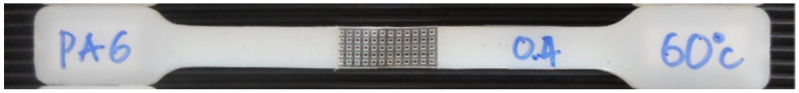
The PA6 material with 0.4 mm mesh thickness.

**Figure 20:**
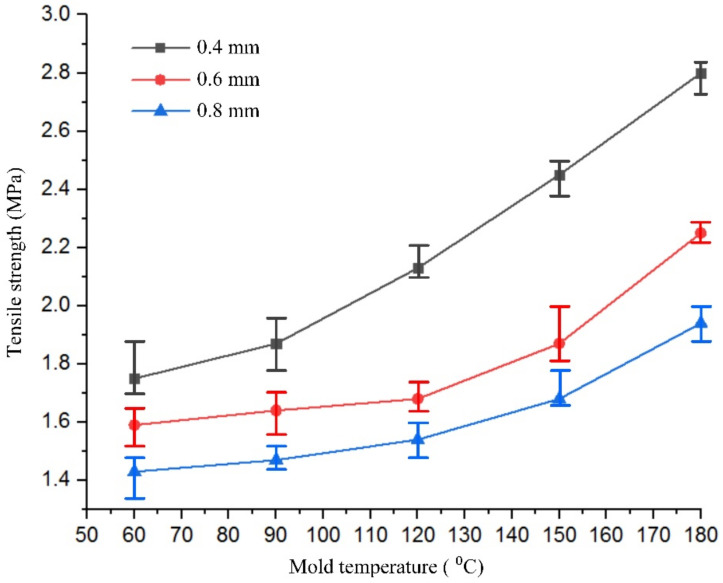
Tensile strength of products with different thicknesses and different mesh area temperatures.

**Figure 21:**
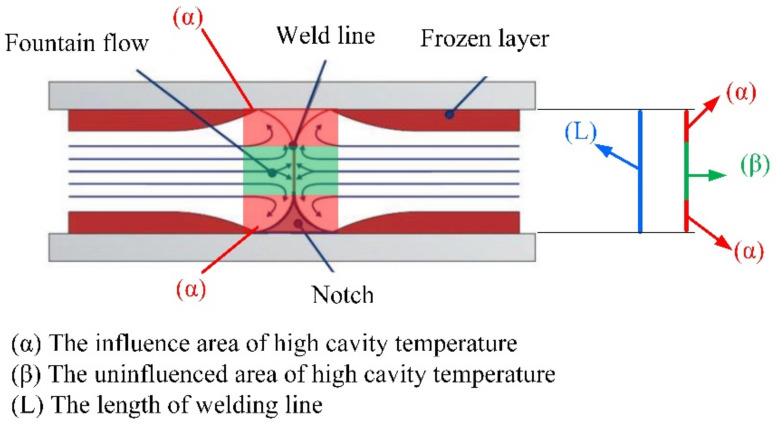
The influence area of high cavity temperature [35].

To study the effect of hot gas heating on the tensile strength of the product, the specimen model in Figure 6 was molded with three mesh thicknesses: 0.4, 0.6, and 0.8 mm. For each type, Ex-GMTC with 400 °C gas was applied to change the mesh area temperature, with a temperature target of 60 to 180 °C. After the injection molding process, the tensile strengths of five samples from each case, as presented in Figure 19, were measured to determine the durability of the product. The Instron-3369 tensile testing machine (Instrom, Norwood, MA, USA) was used for the tensile testing according to the ASTM standard D882 with a testing speed of 12.5 mm/min and a testing temperature of 25 °C. Figure 20 depicts the results of the tensile strength test. Apparently, as the temperature of the mesh area increased from 60 to 180 °C, the tensile strength of the product improved for all thicknesses. With the same part thickness, the higher the cavity temperature, the easier the melt flow and the longer the time required for maintaining the packing pressure at the weld line position. Other studies proved that the packing pressure is the parameter most influencing the weld line tensile strength. Higher packing pressure resulted in increased density and degree of crystallinity [34]. When the packing pressure increased, better molecular bonding and higher molecules concentration occurred, which can be explained by the increase in the tensile strength. Focussing on the length of the weld line as shown in Figure 21, the area influenced by high cavity temperature (α) will be the main position with improved tensile strength. Such improvement was more distinct with the thinner parts, especially with the 0.4 mm thickness, whose tensile strength rose from 1.75 to 2.80 MPa for the target temperature range. With the thinner part, the influence of the hot cavity surface was stronger than for the thicker part. Based on the length of the weld line (Figure 21), the rate of the influence area of high cavity temperature (α) and the length of weld line (L) increased with the thinner product. As a result, higher tensile strength was attained with the thinner part.

## 4. Conclusions

In this study, separate simulations and experiments were conducted to evaluate the weld line appearance and the tensile strength of injection molding products. The findings can be generalized as follows:

- The temperature distribution simulation results indicated that the higher surface temperature appears near the gas gate. The temperature differences were stronger at the heating starting due to the high heating rate at the gas gate area. This phenomenon was observed for all gas temperatures.
- For gas temperatures of 200 to 400 °C, the first 20 s of heating period has high efficiency. With the 400 °C gas, the heating speed was 19.6 °C/s. After 20 s for heating, the temperature of cavity surface got a limitation. In this case, the insert surface reached 158.6 °C. This mold temperature was appropriately high for reducing the frozen layer of almost the plastic materials.
- Employing Ex-GMTC to the 0.4 mm part thickness improved the weld line appearance of the part. In particular, the weld line appearance diminished with an increase in cavity temperature. At 150 °C, the weld line almost disappeared.
- In the tensile strength test, for mold temperatures of 60 °C to 180 °C, the tensile strength of the product improved with the different mesh thicknesses. The empirical results revealed that the percentage increase in tensile strength was greater with thinner parts.
- For future research, Ex-GMTC should be applied simultaneously to both the cavity and core plate. In addition, the heating efficiency could be improved using a fluid controller for hot air, which could reduce the amount of thermal energy that dissipates with the hot air in the environment. For estimating the heating efficiency, the thermal energy should be compared between different heating models to demonstrate the advantages of Ex-GMTC.

## Figures and Tables

**Table 1:** Simulation parameters for studying the heating step.

Parameters	Unit	Value					
Hot air temperature	°C	30	200	250	300	350	400
Density of hot air [33]	kg/m^3^	1.164	0.764	0.675	0.606	0.570	0.524
Heat capacity of hot air [33]	J/kg·K	1004	1026	1035	1046	1057	1068
Thermal-expansion coefficient of hot air [33]	×10^−3^/K	3.32	2.1	1.93	1.76	1.64	1.52
Air pressure	MPa	0.1					
Aluminum density—ASTM B209-14	kg/m^3^	2702					
Heat capacity of aluminum—ASTM B209-14	J/kg·K	903					
Thermal conductivity of aluminum—ASTM B209-14	W/m·K	237					
Simulation type		Transient					

**Table 2:** Molding parameters for the weld line appearance experiment.

Molding Parameter for Polyamide 6 (Lanxess, Durethan B 30 S 000000, non-Reinforced, ISO 1874-PA 6, GR, 14-030, Cologne, Germany)	
Melt temperature	230 °C
Injection pressure	6.86 MPa
Injection time	0.5 s
Packing pressure	5.88 MPa
Packing time	1.5 s
Mold temperature	30 °C
Temperature at the weld line area	30, 60, 90, 120, or 150 °C
Product thickness	0.4 mm
